# P-199. Molecular Epidemiology of the Hepatitis C Virus from the Philippines National Institutes of Health from 2017 to 2025

**DOI:** 10.1093/ofid/ofaf695.421

**Published:** 2026-01-11

**Authors:** Mary Grace T Hernaez, Coleen Pangilinan, Brian Schwem, Ma Jowina Galarion, Angela Salvana, Edsel Maurice Salvana

**Affiliations:** Institute of Molecular Biology and Biotechnology, National Institutes of Health, University of the Philippines Manila, Manila, National Capital Region, Philippines; Institute of Molecular Biology and Biotechnology, National Insitutes of Health, University of the Philippines Manila, Manila, National Capital Region, Philippines; University of the Philippines Manila, Cheyenne, Wyoming; Institute of Molecular Biology and Biotechnology, National Insitutes of Health, University of the Philippines Manila, Manila, National Capital Region, Philippines; Institute of Molecular Biology and Biotechnology, National Insitutes of Health, University of the Philippines Manila, Manila, National Capital Region, Philippines; Institute of Molecular Biology and Biotechnology, National Institutes of Health, University of the Philippines, Manila, National Capital Region, Philippines

## Abstract

**Background:**

Hepatitis C virus (HCV) infection in the Philippines has a prevalence of < 1% and is much less common than Hepatitis B, which has a nationwide prevalence of 10-17%. HCV genotyping is only done sporadically, usually during outbreak investigations, and there is limited surveillance data in literature. Most local HCV infection has been reported among people who inject drugs (PWID), with a co-infection rate of up to 70% among those PWID with HIV. Our central laboratory at the University of the Philippines National Institutes of Health (UP-NIH) is one of a few facilities nationwide that routinely performs HCV genotyping. We report our experience since we started HCV genotyping in 2017 until 2025 to better understand the evolving local molecular epidemiology of HCV.

**Figure 1.**
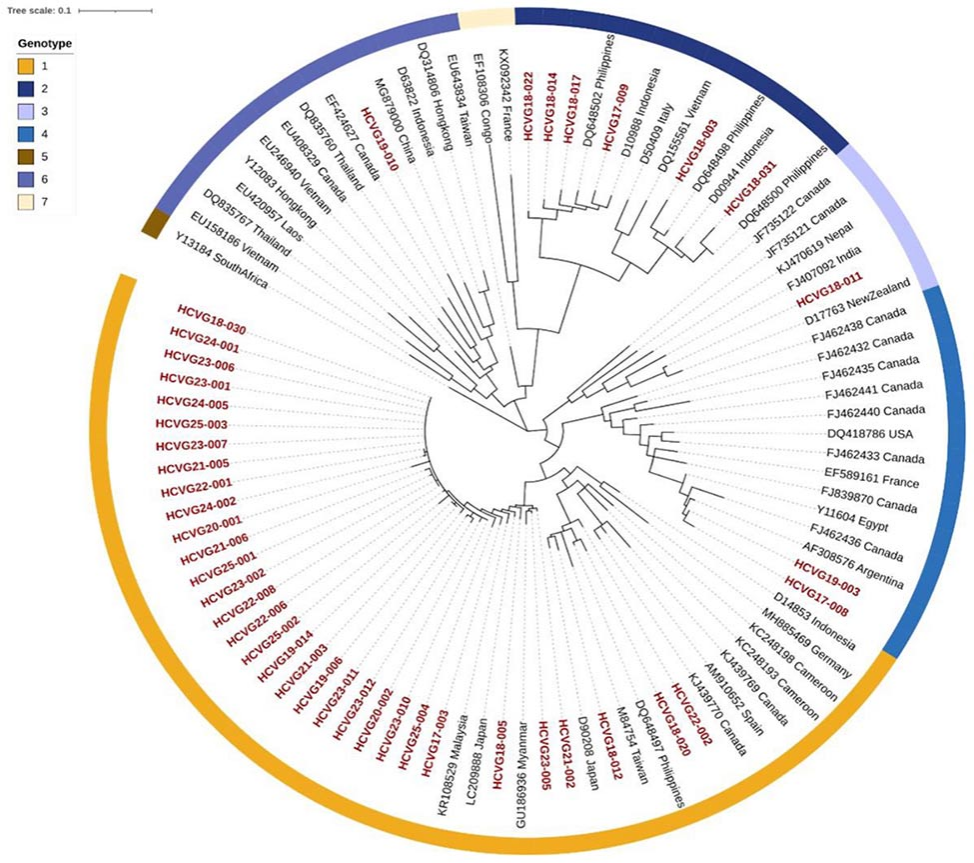
Phylogenetic analysis of HCV genotypes from UP-NIH from 2017 to 2025.

**Methods:**

De-identified HCV-positive clinical plasma samples collected from 2017-2025 and banked at the UP-NIH Central Laboratory were included in the study. This study was exempted from ethical review by the University of the Philippines Manila - Research Ethics Board since no identifying data was collected. The core region (360 bp) was amplified using nested PCR and amplicons underwent capillary sequencing. Sequence quality control and multiple sequence alignment of all sample consensus sequences with representative sequences of major HCV genotypes from the GenBank database were performed using MEGA 7.0. The maximum likelihood tree was constructed with GTR+F+I+G4 model and 1000 bootstrap replicates using IQ-TREE.

**Results:**

A total of 82 samples were analyzed during the study period. Table 1 shows the overall genotype distribution and breakdown across different years. Figure 1 shows the phylogenetic analysis among representative genotypes.

**Table 1.** HCV genotype distribution from 2017 to 2025 at the UP-NIH Central Laboratory.

Year	HCV Genotype							Total per year
	1a	1b	2a	2b	3a	4a	6	
2017	8	1	0	2	0	1	1	13
2018	14	4	4	4	1	0	0	27
2019	12	0	0	1	0	1	1	15
2020	2	0	0	0	0	0	0	2
2021	5	0	0	0	0	0	0	5
2022	3	1	0	0	0	0	0	4
2023	9	0	0	0	0	0	0	9
2024	3	0	0	0	0	0	0	3
2025	4	0	0	0	0	0	0	4
Total (%)	60	6	4	7	1	2	2	N=82

**Conclusion:**

Genotype 1a remains the most prevalent HCV genotype in the Philippines, with no other HCV genotypes other than 1b reported since 2020. This may reflect the increasing uptake of curative treatment globally with the resultant loss in diversity.

**Disclosures:**

All Authors: No reported disclosures

